# Linkage disequilibrium suggests genomic stability in Omicron clades of SARS-CoV-2 from the ASEAN countries

**DOI:** 10.1093/jtm/taad020

**Published:** 2023-02-16

**Authors:** Noriah Binti Mohd Yusof, Zhi Shan Khor, Rehan Shuhada Binti Abu Bakar, Kamal Hisham Bin Kamarul Zaman, Yu Kie Chem, Nur Aina Fatini, Nur Hazliza Binti Salleh, Selvanesan Sengol, Savuth Chin, Sitha Prum, Visal Chhe, Phally Vy, Aizzuddin Mirasin, Nur Amirah Ibarahim, Izzati Azhar, Muhd Haziq Fikry Abdul Momin, Nor Azian Hafneh, Hartanti Dian Ikawati, Hana Apsari Pawestri, Arie Ardiansyah Nugraha, Kartika Dewi Puspa, Archawin Rojanawiwat, Pilailuk Akkapaiboon Okada, Siripaporn Phuygun, Thanutsapa Thanadachakul, Pakorn Piromtong, Hoang Vu Mai Phuong, Ung Thi Hong Trang, Nguyen Phuong Anh, Nguyen Vu Son, Le Thi Thanh, Noorliza Mohamad Noordin, Joon Liang Tan

**Affiliations:** National Public Health Laboratory, Ministry of Health, Putrajaya, Malaysia; Faculty of Information Science and Technology, Multimedia University, Melaka, Malaysia; National Public Health Laboratory, Ministry of Health, Putrajaya, Malaysia; National Public Health Laboratory, Ministry of Health, Putrajaya, Malaysia; National Public Health Laboratory, Ministry of Health, Putrajaya, Malaysia; National Public Health Laboratory, Ministry of Health, Putrajaya, Malaysia; National Public Health Laboratory, Ministry of Health, Putrajaya, Malaysia; National Public Health Laboratory, Ministry of Health, Putrajaya, Malaysia; National Public Health Laboratory, National Institute of Public Health, Phnom Penh, Cambodia; National Public Health Laboratory, National Institute of Public Health, Phnom Penh, Cambodia; National Public Health Laboratory, National Institute of Public Health, Phnom Penh, Cambodia; National Public Health Laboratory, National Institute of Public Health, Phnom Penh, Cambodia; Department of Laboratory Services, Ministry of Health, Brunei; Department of Laboratory Services, Ministry of Health, Brunei; Department of Laboratory Services, Ministry of Health, Brunei; Department of Laboratory Services, Ministry of Health, Brunei; Department of Laboratory Services, Ministry of Health, Brunei; National Referral Laboratory Prof. Sri Oemijati, Center for Resilience and Human Resources, Health Policy Agency, Jakarta, Indonesia; National Referral Laboratory Prof. Sri Oemijati, Center for Resilience and Human Resources, Health Policy Agency, Jakarta, Indonesia; National Referral Laboratory Prof. Sri Oemijati, Center for Resilience and Human Resources, Health Policy Agency, Jakarta, Indonesia; National Referral Laboratory Prof. Sri Oemijati, Center for Resilience and Human Resources, Health Policy Agency, Jakarta, Indonesia; Department of Medical Sciences, Ministry of Public Health, National Institute of Health, Yasothon, Thailand; Department of Medical Sciences, Ministry of Public Health, National Institute of Health, Yasothon, Thailand; Department of Medical Sciences, Ministry of Public Health, National Institute of Health, Yasothon, Thailand; Department of Medical Sciences, Ministry of Public Health, National Institute of Health, Yasothon, Thailand; Department of Medical Sciences, Ministry of Public Health, National Institute of Health, Yasothon, Thailand; National Institute of Hygiene and Epidemiology (NIHE), Ðà Nãng, Vietnam; National Institute of Hygiene and Epidemiology (NIHE), Ðà Nãng, Vietnam; National Institute of Hygiene and Epidemiology (NIHE), Ðà Nãng, Vietnam; National Institute of Hygiene and Epidemiology (NIHE), Ðà Nãng, Vietnam; National Institute of Hygiene and Epidemiology (NIHE), Ðà Nãng, Vietnam; National Public Health Laboratory, Ministry of Health, Putrajaya, Malaysia; National Institute of Hygiene and Epidemiology (NIHE), Ðà Nãng, Vietnam; Faculty of Information Science and Technology, Multimedia University, Melaka, Malaysia

##  

After more than 2 years of pandemic caused by SARS-CoV-2, COVID-19 is still a national concern in many countries worldwide. One of the key investigations is to understand the factors contributing to the evolutionary dynamics of SARS-CoV-2 as a pathogen. Currently, almost all countries have lifted border control orders and have allowed inter-country travel with minimal restrictions. This provides better resolutions on genomic patterns and the evolution of circulating SARS-CoV-2 in each community with the influence of imported strains.

In this report, we surveyed genomes of SARS-CoV-2 strains circulating in the Association of Southeast Asian Nations (ASEAN) countries. This project serves as a collaborative effort from the ASEAN Member States that had participated in the programme ‘Strengthening Laboratory Capacity on COVID-19 Bio Genomic for ASEAN Countries’. A total of 124 SARS-CoV-2 samples were collected by the national level public health laboratories: Malaysia (*n* = 24), Brunei Darussalam (*n* = 20), Cambodia (*n* = 20), Indonesia (*n* = 20), Thailand (*n* = 20) and Vietnam (*n* = 20). All samples were sequenced using short-read technology. The version of the genomes used in this study was statistically quality-assessed and preprocessed in FASTQC and Trimmomatics, respectively, prior to *de novo* assembly in Megahit.^1–3^ The genomes were submitted to GISAID by the respective laboratories (Supplementary Data 1).

**Figure 1:**
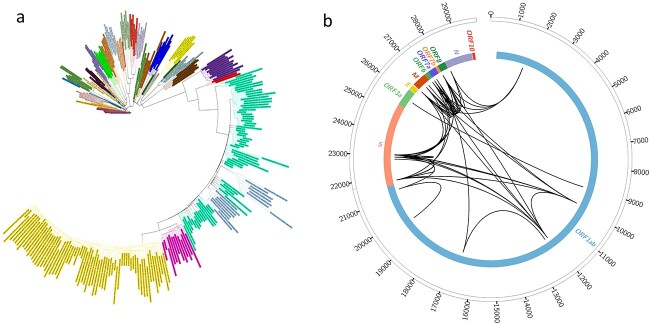
(a) Unrooted representation for phylogenomics of 124 genomes yielded from this study with 210 NextStrain clade representative genomes. Each colour represented single clade. (b) Illustration of linkage disequilibrium in the SARS-CoV-2 genomes.

Phylogenomics analyses using 210 representative genomes from the NextStrain database (accessed on 25 August 2022) as background, assigned all the genomes into four different clades in the Omicron lineage. The majority of the strains were clustered into 22B (48.39%), followed by 22L (44.35%), 22A (4.03%) and, lastly, 21K (3.23%). Each strain was clustered into a respective clade and showed a non-observable cascade-like structure, indicating the genomes of SARS-CoV-2 exerted a slow impact on evolution (Figure 1).

There are speculations on the roles of recombination in viruses, such as mechanisms for rapid repair and host adaptation, as well as viral genome integrity.^4^ In this study, there were no observable recombination trends in the reconstructed phylogenomics trees. Thus, the hypothesis of recombination in the SARS-CoV-2 was further tested with inter- and intra-clades PHI test of recombination. Both tests showed insignificant recombination events among the strains at a *P*-value of 0.405 and 0.253, respectively.

Linkage disequilibrium measures the non-random association of nucleotides at different sites. Analyses of linkage disequilibrium have been reported to be able to infer evolutionary features in pathogens.^5^ In this study, linkage disequilibrium analyses were performed on the genomes of the SARS-CoV-2 analysed (Table 1). Using a threshold of 0.8 for a correlation between alleles at two loci (*R*^2^), 22 sites were predicted to be under the effect of linkage disequilibrium. All the sites achieved high-confidence disequilibrium coefficients (D′) ranging from 0.96 to 1.0, which were supported by Fisher’s Exact test ranging from 3.65 × 10^−37^ to 1.06 × 10^−10^. One linkage disequilibrium was found to involve 13 out of the 22 predicted sites (LD Set 1). The sites involved 11 SNP-SNP, single SNP-INDEL and single triplet-nucleotide linkage disequilibria. The linkage disequilibrium for the remaining sites involved only SNP-SNP associations, with four (LD Set 2), three (LD Set 3) and two (LD Set 4) affected sites, respectively. Recombination processes were found to disrupt linkage disequilibrium and managed to alter the variants-associated sites.^6^ In this study, the positions of the linkage disequilibrium pairs ranged from 22 to 26 712 bp (Figure 1). Coupled with the PHI test of recombination, the close-to-wide pairing distances of linkage disequilibrium suggested that the genomes of SARS-CoV-2 had little to no recombination influence.

**Table 1:** Details of linkage disequilibrium

	Sites	LD #1	LD #2	LD #1 Codon = Amino	LD #2 Codon = Amino	Gene
	1627	T	C	CT[T] = L	CT[C] = L	*nsp2*
	9866	C	T	[C]TT = L	[T]TT = F	*nsp4*
	12 160	A	G	GA[A] = E	GA[G] = E	*nsp8*
LD Set 1				IHVdel	A[TA] = I [CAT] = H [G]TC = V	
	21 765–21 770	–	tacatg			*S*
				
	22 917	G	t	C[G]G = R	C[T]G = L	*S*
	23 018	G	t	[G]TT = V	[T]TT = F	*S*
	23 040	A	G	C[A]A = Q	C[G]A = R	*S*
	26 529	A	G	[A]AT = N	[G]AT = D	*M*
	26 858	C	T	TT[C] = F	TT[T] = F	*M*
	27 259	A	C	[A]GG = R	[C]GG = R	*ORF6*
	27 382–27 384	GAT	CTC	GAT = D	CTC = L	*ORF6*
	27 889	T	C	–	–	–
	28 330	G	A	GG[G] = G	GG[A] = G	*N*
	12 310	A	G	CA[A] = Q	CA[G] = Q	*nsp8*
LD Set 2	16 616	A	C	A[A]T = N	A[C]T = T	*nsp13*
	27 012	T	C	[T]TG = L	[C]TG = L	*M*
	27 513	T	C	TA[T] = Y	TA[C] = Y	*ORF7a*
LD Set3	19 677	T	G	CA[T] = H	CA[G] = Q	*nsp15*
	21 306	T	C	CG[T] = R	CG[C] = R	*nsp16*
	22 812	C	A	A[C]G = T	A[A]G = K	*S*
LD Set 4	8991	T	C	G[T]A = V	G[C]A = A	*nsp4*
	25 810	T	C	[T]TT = F	[C]TT = L	*ORF3a*

Note: The genomes will either have LD Pattern 1 or LD Pattern 2 across the positions in each LD set.

Successful reproduction in a population is the indicator of genome stability. Recombination is one of the common processes used for viral genome repair and can introduce mutations into the host (reviewed by Kockler and Gordenin).^7^ Hence, the process increases the diversity in a population. However, it has been reported that there is a lack of genomic diversity (low frequency of nucleotide changes) among SARS-CoV-2 strains.^8^ Although the recombination in SARS-CoV-2 has been discussed in multiple publications,^9^ the number of recombinant strains has not been found to be alarming. It was reported that only approximately 2–3% out of the total SARS-CoV-2 genomes deposited in the public database exerted recombination events.^10^ The recombinant strains were also predicted to be present in the population only for a short period of time. Together with all these aspects, a strong influence of linkage disequilibrium from our study suggests the stability of Omicron clades SARS-CoV-2 genomes. The stability in the genomes indicates that random nucleotide changes are less likely to occur in the SARS-CoV-2. Nucleotide changes, especially linkage disequilibrium, can have a direct influence on the virulence and vaccine effectiveness in the infected hosts. This characteristic of SARS-CoV-2 will raise public health concerns if new variants are identified. Further studies are needed to evaluate the linkage disequilibrium among all SARS-CoV-2 clades and the impact on their fitness factor. This study also provides better insights for other researchers to thoroughly customize specific parameters to analyse the trend of infection and drug design in combating SARS-CoV-2.
